# Development and Validation of an LC-MS/MS Method for the Simultaneous Determination of Alprazolam, Bromazepam, Clonazepam, Diazepam and Flunitrazpam in Human Urine and Its Application to Samples from Suspected Drug Abusers

**DOI:** 10.3390/molecules30173451

**Published:** 2025-08-22

**Authors:** Husein Kamal, Varun Gandhi, Lina Akil, Naser F. Al-Tannak, Nicholas J. W. Rattray, Ibrahim Khadra

**Affiliations:** 1 Strathclyde Institute of Pharmacy and Biomedical Sciences, University of Strathclyde, 161 Cathedral Street, Glasgow G4 0RE, UK; husein.kamal@strath.ac.uk (H.K.); varun.gandhi.2022@uni.strath.ac.uk (V.G.); lina.akil@strath.ac.uk (L.A.); nicholas.rattray@strath.ac.uk (N.J.W.R.); 2 Department of Pharmaceutical Chemistry, Faculty of Pharmacy, Kuwait University, P.O. Box 24923, Safat 13110, Kuwait; dr.altannak@ku.edu.kw

**Keywords:** benzodiazepines, drug abuse, LC-MS/MS, urine analysis, diazepam

## Abstract

A simple and reliable method was developed using LC-MS/MS to quantify alprazolam, bromazepam, clonazepam, diazepam, and flunitrazepam in clinical samples. This method was validated for the simultaneous determination of alprazolam, bromazepam, clonazepam, diazepam, and flunitrazepam. It was applied to human urine samples collected from people suspected of drug abuse in the Kuwaiti region. Formic acid in water and acetonitrile was used in mobile phase with a gradient mode of elution using C18 reverse-phase column. The instrument was operated in a positive mode with an electrospray ionization source using multiple reaction monitoring. For sample extraction, the liquid-liquid extraction technique was used. The method was validated for limit of detection, limit of quantitation, selectivity, linearity, accuracy, and precision. The concentration for limit of quantitation was 6.0 ng/mL, the linearity ranged from 2.0 to 300 ng/mL for each of the analytes, and the r^2^ values were ≥0.99. The accuracy was found to be within a range of 80–120% and precision had a %RSD of ≤15% for each of the analytes. The method was applied to 48 urine samples collected from those suspected of drug abuse by the Toxicology Department of the General Department of Criminal Evidence, Kuwait, and alprazolam, bromazepam, clonazepam, diazepam and flunitrazepam were identified commonly in the samples. The overall drug positivity rate obtained considering 48 samples was 93.75%. Based on these results and successful determination of alprazolam, bromazepam, clonazepam, diazepam and flunitrazepam in human urine samples from those suspected of drug abuse, this method is deemed to be suitable for its routine analysis.

## 1. Introduction

Benzodiazepines have been widely used for decades in the treatment of anxiety, depression, insomnia, and seizure-related disorders [1]. These drugs exert their therapeutic effects by acting on gamma-aminobutyric acid-A (GABAA) receptors in the brain, resulting in moderate to profound central nervous system (CNS) depression [1]. However, their use is often accompanied by side effects such as drowsiness, nausea, weakness, headache, confusion, loss of appetite, impaired motor coordination, and short-term memory loss [1].

Due to their psychoactive effects, benzodiazepines are frequently misused for their euphoric properties or in criminal activities involving drug-facilitated offences in certain regions [1]. In 2018, Kuwait, a country in the Middle East and part of the MENA region, reported that approximately 43% of forensic toxicology cases involved benzodiazepine abuse [2]. The Narcotic and Psychotropic Laboratory of Kuwait, under the General Department of Criminal Evidence, identified alprazolam, bromazepam, clonazepam, diazepam, and flunitrazepam as the most commonly encountered benzodiazepines in such cases [3].

To confirm cases of drug abuse or addiction, it is essential to detect traces of these drugs in forensic samples using highly sensitive analytical techniques. Liquid chromatography coupled with tandem mass spectrometry (LC-MS/MS) has emerged as a preferred method due to its sensitivity and specificity [3]. This study presents the development and validation of a rapid, simple, and sensitive LC-MS/MS method for the simultaneous determination of alprazolam, bromazepam, clonazepam, diazepam, and flunitrazepam in human urine samples.

Urine is the preferred specimen in forensic toxicology due to its non-invasive collection, availability of FDA-approved screening kits, and higher concentration of drugs and their metabolites compared to blood or plasma. Urine also offers a broader detection window [4]. It is especially useful in cases involving behavioral or mental status changes or for monitoring drug abstinence among young adults [5]. However, urine analysis has limitations, including a narrower detection window for some drugs and poor correlation with systemic drug concentrations compared to blood or plasma samples [4].

Various LC-MS/MS methods have been developed for the detection of benzodiazepines in biological matrices such as plasma [6,7,8,9,10,11], urine [12,13,14,15,16], combined plasma and urine [1,17], oral fluids [18,19], and hair [13,15,16,20,21,22]. Although gas chromatography–mass spectrometry (GC-MS) has also been used, it typically requires derivatization of benzodiazepines before analysis [23,24,25,26,27]. In contrast, LC-MS/MS eliminates this additional step, making it more practical and efficient. High-performance liquid chromatography with UV detection (HPLC-UV) has also been employed for plasma analysis of certain benzodiazepines [28,29,30], but no validated LC-MS/MS method exists specifically for the simultaneous quantification of alprazolam, bromazepam, clonazepam, diazepam, and flunitrazepam in human urine.

Enzyme immunoassays (EIAs) are commonly used for initial drug screening due to their cost-effectiveness and rapid results. However, they lack the sensitivity and specificity needed for definitive identification and quantification [1,31]. In the past, GC-MS technology was the preferred method for drug confirmation; however, it often involves time-consuming sample preparation and might require solid-phase extraction. Moreover, for non-volatile or thermolabile compounds, derivatization is necessary, and the technique is further limited by the lengthy GC cycle time [23,24,32]. With recent advancements in analytical technology, LC-MS/MS has become the gold standard for confirmatory drug testing in forensic laboratories [4].

Considering MS detector, several factors such as the accuracy of calibrator concentrations can influence the quantification accuracy of an assay. A factor specific to MS detection is the matrix effect, which refers to ionization suppression or enhancement caused by co-eluting compounds present in biological specimens [33,34]. LC-MS/MS is generally more susceptible to matrix effects than GC-MS [32,35]. These effects can be assessed through various experimental approaches, and several strategies such as the use of stable isotope-labeled internal standards can be employed to minimize their impact [32,36]. Still, the matrix effect observed than specimen sample shall be subjected to cleaning of matrix using the dispersive liquid–liquid microextraction (DLLME) approach [37,38].

In forensic toxicology, liquid chromatography-tandem mass spectrometry (LC-MS/MS) [11,15,16,18,19,20,22,23,24] is increasingly preferred over gas chromatography-mass spectrometry (GC-MS) [25,26,27] for drug screening of suspects using various biological matrices, i.e., urine hair and blood. LC-MS-MS is an efficient and practical alternative for benzodiazepine confirmation testing due to its rapid and simple sample extraction, the ability to analyze a wider variety of compounds, and shorter run times across different specimen types collected from forensic cases [11,15,16,18,19,20,22,23,24]. High-throughput dispersive liquid–liquid microextraction (DLLME) is an effective technique for purifying specimens such as blood and urine, allowing for the simultaneous extraction of multiple drug classes such as benzodiazepines, antipsychotic drugs from the specimens using minimal volumes of organic solvents for LC-MS/MS [37], capillary electrophoresis with time of flight mass spectrometry [38], HPLC with UV detection [39,40,41], and GC-MS [42] with sample derivatization following DLLME extraction.

The issue of benzodiazepine abuse is not confined to Kuwait. The European Drug Report (2019) noted a rising trend in the use of benzodiazepines, with many new compounds lacking international regulatory control. Between 2014 and 2019, 28 new benzodiazepines were monitored and 23 were detected across Europe, raising significant public health concerns [43]. With the above rationale, this study aims to develop and validate a simple, sensitive, and rapid LC-MS/MS method for the simultaneous determination of alprazolam, bromazepam, clonazepam, diazepam, and flunitrazepam in human urine, and to apply this method to forensic samples from those suspected of drug abuse.

## 2. Results and Discussion

### 2.1. Method Development

**Figure 1:**
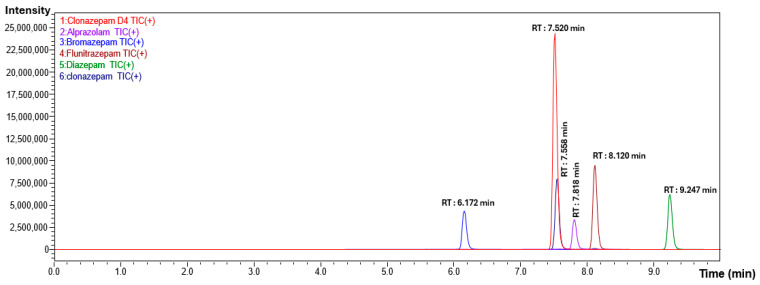
Chromatogram of mix standard at 100.0 ng/mL and internal standard at 100.0 ng/mL.

**Figure 2:**
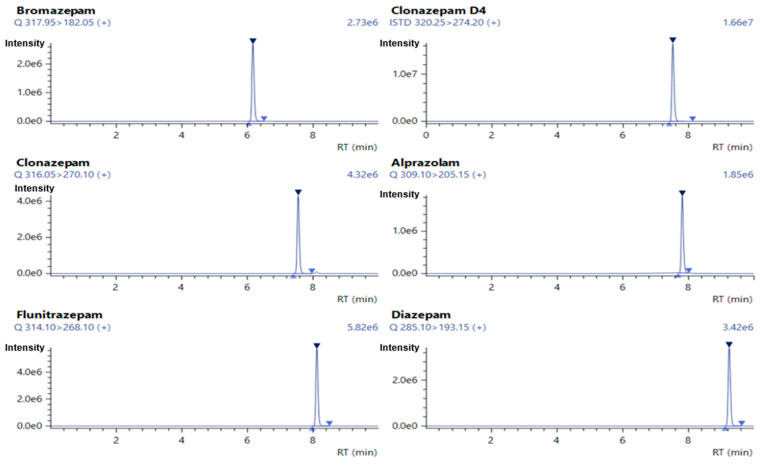
The retention time of bromazepam, clonazepam-d4, clonazepam, alprazolam, flunitrazepam and diazepam are 6.17, 7.52, 7.55, 7.81, 8.12 and 9.24 min, respectively.

Based on the nature of compounds under analysis, reverse-phase chromatography was found suitable and used as a separation technique. In presence of polar mobile phase with 0.1% *v/v* formic acid, the compound under analysis becomes completely or partially ionized as per their respective pKa and tends to be partitioned on the non-polar C18 stationary phase, which results in good chromatographic separation. The mobile phase A consists of 0.1% *v/v* formic acid in 5% acetonitrile and mobile phase B consists of 0.1% *v/v* formic acid in 95% acetonitrile as a gradient to maintain the polarity of mobile phase [14]. The optimized flow rate for the gradient was 0.6 mL/min, which results in well-resolved peaks. The resulting chromatogram obtained is shown in Figure 1 and Figure 2 for alprazolam, bromazepam, clonazepam, flunitrazepam, diazepam and clonazepam-d4 with well-separated, sharp distinct peaks along with good response from the developed chromatographic conditions with the run time of 15 min.

### 2.2. Method Validation

The developed method was validated as per ICH guideline Q2(R1) [44], and the obtained results are discussed below.

#### 2.2.1. Specificity

The chromatogram obtained from blank sigmatrix urine diluent were compared with spiked sigmatrix urine diluent at the limit of quantitation (LOQ) for each of the analytes along with internal standard and diluent to identify the interference due to biological matrix. No interference was observed in the retention time in minutes for bromazepam, clonazepam-d4, clonazepam, alprazolam, flunitrazepam and diazepam in the sigmatrix urine diluent blank, and results were found within the limit of response of not more than 20% of the LOQ in the blank sigmatrix urine diluent for analytes. Hence, this method was found to be selective for the quantification of bromazepam, clonazepam-d4, clonazepam, alprazolam, flunitrazepam and diazepam.

#### 2.2.2. Linearity and Calibration Curve

The linearity was determined at *n* = 3 for each level over the range of 2.0–300.0 ng/mL for bromazepam, clonazepam, alprazolam, flunitrazepam, diazepam and clonazepam-d4 as an internal standard with concentration of 100.0 µg/mL; the linear fit was observed for the calibration curve when plotted for mean peak area ratio of standard versus its concentration. The regression equation observed was y = 0.049x − 0.004, r^2^ = 0.9985 for bromazepam; y = 0.047x + 0.927, r^2^ = 0.9990 for clonazepam B2; y = 0.019x − 0.007, r^2^ = 0.9987 for alprazolam; y = 0.026x + 0.000, r^2^ = 0.999 for flunitrazepam; and y = 0.026x + 0.000, r^2^ = 0.9997 for diazepam. The r^2^ value obtained for all the analytes under analysis were found well within the limit of r^2^ ≥ 0.99; hence, it is considered as linear over the calibration range.

#### 2.2.3. Limit of Quantification and Limit of Detection

The LOQ (*n* = 6) was established at 6.0 ng/mL with %RSD of 2.27%, 1.20%, 1.98%, 1.50% and 0.12%; LOD (*n* = 3) was established at 2.0 ng/mL with %RSD of 1.85%, 3.05%, 2.38%, 2.43% and 1.21% for bromazepam, clonazepam, alprazolam, flunitrazepam and diazepam, respectively. The %RSD should be no more than 20.0%. Based on the results, it was concluded that LOQ along with LOD was established at 6.0 ng/mL and 2.0 ng/mL, respectively.

#### 2.2.4. Accuracy

The results for within-run accuracy are shown in Table 1. The results for accuracy at each level were within the limit of ±20% of the nominal value, and it was concluded that it meets the acceptance criteria for accuracy. The %RSD results for each level were below 15.0%. Hence, with these results, this method is considered accurate.

#### 2.2.5. Precision

The results of precision, repeatability, and intermediate precision are shown in Table 2. The results for %RSD of peak area ratio should not be more than 2.0%. The results obtained were well within the limit. From these, it is concluded that this method is precise.

Overall, the method was successfully validated and results were acceptable. Additionally, comparing our overall method to other published methods such as that of Sofalvi S et al., our method has the simplest preparation using liquid-liquid extraction, the mobile phase used does not require addition of salt or pH adjustment as the pH is controlled directly with the exact amount of formic acid, and the results are accurate and precise. Comparing our method to the quantitation of analysed compounds in urine matrix with oral fluid, which was estimated by Jang M et al., and in whole blood, as estimated by Smink BE et al., similar quantitation range and recovery were obtained, which were equivalent to their biological matrix, which is advantageous to this method. ChèzeM et al. determined levels of bromazepam and clonazepan from urine samples, and our method’s sensitivity was almost the same, as they established LLOQ at 1 ng/mL and we established LLOQ at 2 ng/mL, which is similar in relation to sensitivity and precision. Glover SJ et al. determined levels of benzodiazepines from urine and screened ELISA samples using LCMS samples. Their method had a drug positive identification rate of 89%, and our method achieved a rate of 93.75%. The LLOQ determined for Diazepam is 5 ng/mL, and we achieved a value of 2 ng/mL for diazepam. Hence, on the whole, our method shows a good level of detection, quantitation, and sensitivity compared to previously published methods.

### 2.3. Application to Samples from Suspected Drug Abusers

The main aim of this study was to scan the Kuwaiti market, identify the common benzodiazepines abused in Kuwait, and quantify their amounts in products sold on the streets. For this purpose, 48 samples of benzodiazepines products were provided by the Toxicology Department of the General Department of Criminal Evidence, Kuwait. The samples were collected from November 2022 to May 2023.

**Figure 3:**
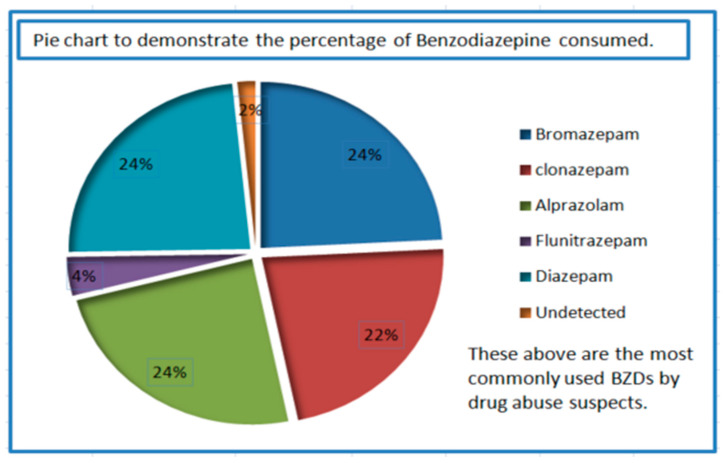
Graphical representation in terms of percentage of benzodiazepines used by the suspects.

**Figure 4:**
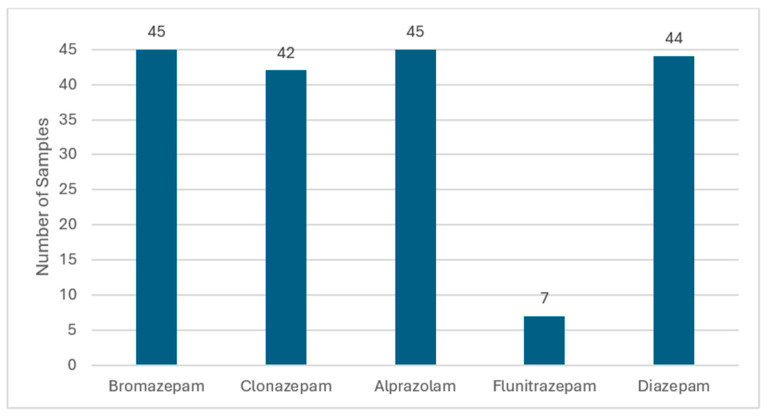
Analysis of urine samples from Kuwait showing the occurrence of each benzodiazepine and their combinations.

The validated LC-MS/MS method was applied to screen 48 samples to identify the benzodiazepines used in the sold products and to quantify their concentrations. Each sample was analyzed thrice, and the average concentrations are listed in Table 3. Considering 48 samples as a 100%, about 93.75% of the samples contained bromazepam and alprazolam, 91.66% contained diazepam, 87.5% contained clonazepam, while 14.58% contained flunitrazepam and 2% of samples did not contain any traces from the benzodiazepines, which indicates the compounds were not detected in the suspects. This suggests that the most common benzodiazepines (BZDs) in the Kuwaiti market were bromazepam and alprazolam, followed by diazepam and clonazepam. The overall drug positive rate obtained considering 48 samples was 93.75%. The quantitative data of the samples indicate that the concentrations of these five benzodiazepines ranged from 0.465 to 233.7 ng/mL (Table 3), and Figure 3 and Figure 4 indicate the percentage and occurrence of benzodiazepines used by the suspects.

This investigation represents the first study to employ a fully validated analytical workflow for the quantification of benzodiazepines in street-acquired urine samples in Kuwait. While the total sample size (*n* = 48) is modest, rigorous method validation confirmed excellent accuracy, precision, sensitivity, and specificity, demonstrating the reliability of the assay. These performance characteristics are critical given the potential for benzodiazepine exposure to precipitate severe adverse effects, including fatal toxicity.

Although the study setting is Kuwait—specifically, urine specimens obtained from the General Department of Criminal Evidence, Ministry of Interior, State of Kuwait—the analytical protocol itself is universally applicable to forensic or clinical toxicology casework, irrespective of sample origin. By eliminating geographic dependency, our methodology establishes a template adaptable to diverse laboratory environments.

Extensions of this research will involve significantly increasing sample throughput, incorporating both urine and blood–plasma matrices, and broadening the panel to include other illicit psychoactive substances frequently co-administered with benzodiazepines. This approach aims to elucidate patterns of poly-substance misuse in Kuwait’s urban setting.

Moreover, planned inclusion of postmortem specimens affords substantial forensic and medico-legal value. Analyses of postmortem matrices will enable identification of benzodiazepine-associated fatalities, support determination of cause of death, refine interpretation of toxicological data in the context of postmortem redistribution and metabolic degradation, and contribute evidentiary value in judicial proceedings.

By emphasizing the scalability, sensitivity, and versatility of the LC-MS/MS technique across ante- and postmortem contexts, the study underlines significant utility in coroner investigations, retrospective surveillance programmes, public health surveillance of drug misuse, and strategic decision-making in forensic toxicology and legal medicine.

## 3. Material and Methods

### 3.1. Chemicals and Reagent

Acetonitrile and Methanol HPLC grade were purchased from VWR chemicals, Leicestershire, UK. Formic acid and Sodium bicarbonate AR grade were purchased from VWR chemicals, Leicestershire, UK. Alprazolam, Bromazepam, Clonazepam, Clonazepam-D4, Diazepam, Flunitrazepam and Sigmatrix urine diluent (synthetic urine) were procured from Sigma Aldrich, Dorset, UK. PURA-Q+20 HPLC grade water from SLS Lab Pro, water purification remote dispenser purchased from SLS scientific laboratories supplies, Nottingham, UK was used.

### 3.2. Instruments and Chromatographic Conditions

**Figure 5:**
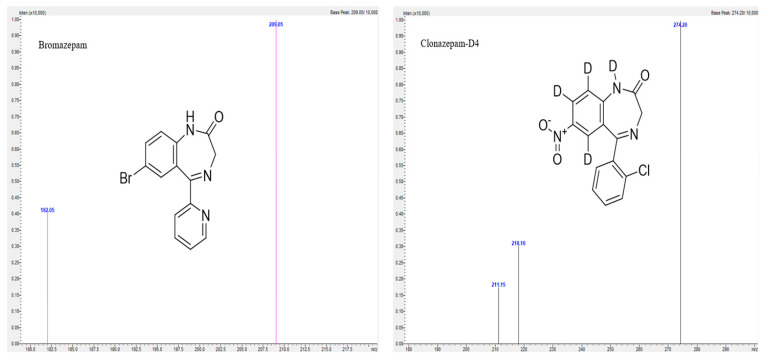
Product ion mass spectra of Bromazepam and Clonazepam-D4.

**Figure 6:**
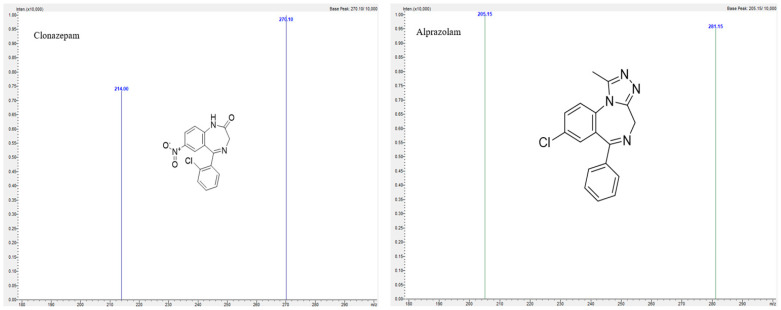
Product ion mass spectra of Clonazepam and Alprazolam.

**Figure 7:**
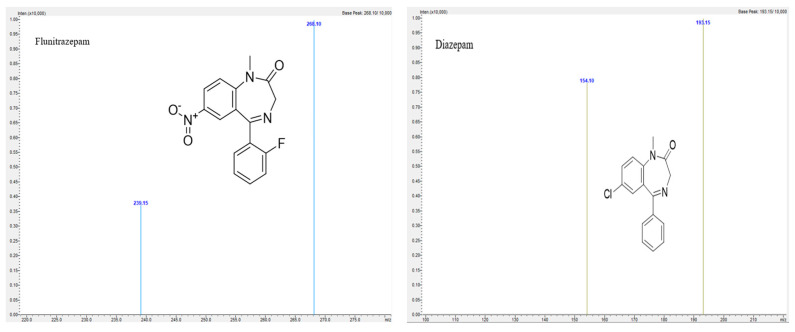
Product ion mass spectra of Flunitrazepam and Diazepam.

For development and validation of the method, Shimadzu LC2040C, Nexera-i, with LCMS-8050 triple quadrupole mass spectrometer purchased from Shimadzu UK Limited, Milton Keynes, UK was used. A C18 ACE column from Merck, 150 × 4.6 mm, 3 μm was used for analysis. Mobile phase was 0.1% *v/v* formic acid in 5% acetonitrile, and 0.1% *v/v* formic acid in 95% acetonitrile was used as mobile phase B in a gradient elution mode (Table 4) with a flow rate of 0.6 mL/min. The column temperature was 50 °C. An amount of 1 µL of sample was injected to the column for analysis, and the samples were kept in a vial tray at 4 °C. For this method, Clonazepam-D4 was used as an internal standard. The LabSolutions, version 5.109 software were used for analysis of chromatograms. Positive electrospray ionization (ESI) mode was used for mass spectrometric analyses. The optimum mass spectrometer parameters for analysis are as follows: interface temperature was 300 °C, desolvation temperature was 526 °C, heating and drying gas flow was 10 L/min. The multiple reaction monitoring (MRM) transitions, retention time (minutes) and collision energy are presented in Table 5. The product ion mass spectra along with structure are given in Figure 5, Figure 6 and Figure 7. The compounds are weakly basic and lipophilic in nature; hence, in order to improve retention time of the analyte, the acidic mobile phase was selected, so that basic compounds become completely or partially ionized and do not spend more time for partitioning in the stationary phase. Likewise, all the compounds elute by 10 min; for the next 2 min, a constant organic flow was maintained to remove any unretained compounds. From 12.10 min, column equilibration started to achieve the initial composition for the next run.

### 3.3. Preparation of Standard Solutions and Samples

#### 3.3.1. Preparation of Standard Solutions

The standard stock solutions of alprazolam, bromazepam, clonazepam-d4, clonazepam and flunitrazepam were prepared in methanol (1000 µg/mL). Furthermore, these standards were diluted with methanol to achieve the final concentration of 2–300 ng/mL for alprazolam, bromazepam, clonazepam and flunitrazepam and a fixed concentration of clonazepam-d4 (an internal standard) at 100 ng/mL throughout the series of standard solutions.

#### 3.3.2. Preparation of Control Sample from Sigma-Trix Urine Diluent

The liquid-liquid sample extraction method was adopted as it is easy to perform and is also cost-effective in comparison to solid-phase extraction; also, it does not require any specific procedural set-up or any cartridge selection and is able to provide the cleaner extract required for this analysis.

To a micro centrifuge tube, 100 µL of sigmatrix urine diluent was added along with 25 µL of clonazepam-d4 solution (4 µg/mL) as the internal standard. To this, 100 µL of sodium bicarbonate of pH 9.5 was added, after which methanol was incorporated to bring the remaining volume up to 1 mL. The contents were mixed by gentle shaking, and then, the tube was vortexed for 30 s, placed on a mechanical shaker for 10 min, and centrifuged for 15 min at 130 × 100 RPM. The supernatant layer was collected and filtered with 0.22 µ PTFE filter and transferred to an HPLC vial and injected.

#### 3.3.3. Preparation of Processed Quality Control Samples

The processing of the quality control samples was performed using liquid–liquid sample extraction. To a micro centrifuge tube, 100 µL of sigma-trix urine diluent wasadded with a mixed standard of alprazolam, bromazepam, clonazepam and flunitrazepam at four concentration levels: 2.0, 6.0, 100.0, 200.0 ng/mL. The samples were processed in the same manner as specified in Section 3.3.2.

### 3.4. Method Validation

The sample preparation for method validation activity was performed as given below.

#### 3.4.1. Specificity

To evaluate specificity, standards of blank sigma-trix urine diluent without spiking the mix were prepared and processed in the same manner as specified in Section 3.3.2. The chromatogram obtained from blank sigma-trix urine diluent were compared with spiked sigma-trix urine diluent at the limit of quantitation (LOQ) for each of the analytes, along with an internal standard, to identify the interference due to matrix.

#### 3.4.2. Linearity

Linearity was evaluated over a series of calibration mix standards, and a calibration curve was plotted for mean peak area ratio of standard versus its concentration. When the r^2^ value is ≥0.99, the method is deemed to be linear.

#### 3.4.3. Limit of Quantification and Detection

LOQ and LOD were established based on a visual evaluation approach using mix standard, and the obtained concentration was applied to sigma-trix urine diluent sample for establishing LOQ. The limit of quantification is the minimum concentration where analytes are reliably quantified with an acceptable accuracy (%Recovery should be within 80–120%) and precision (%RSD should be within 20%). The spiked samples were prepared as mentioned in Section 3.3.3.

#### 3.4.4. Accuracy

To evaluate the within-run accuracy, the sigma-trix urine diluent was processed 3 times in a different preparation (*n* = 3) as mentioned in Section 3.3.3, at four different quality control sample levels (2.0, 6.0, 100.0 and 200.0 ng/mL).

#### 3.4.5. Precision

To evaluate the precision, repeatability and intermediate precision were evaluated on the mix standard and internal standard using 100.0 ng/mL solutions. The intermediate precision was evaluated on the next day of the repeatability parameter.

### 3.5. Application to Samples from Suspected Drug Abusers

Sample collection procedure: Urine samples were collected from those suspected of drug abuse or those who were arrested or inspected by a policeman. The samples were labeled and then sent to a forensic laboratory. The samples were collected systematically and stored in −20 °C until the results were out. The Toxicology Department of the General Department of Criminal Evidence in Kuwait supplied 48 samples seized from Kuwaiti streets between November 2022 and May 2023. From each sample, 100 µL of human urine diluent was added along with 25 µL of clonazepam-d4 solution (4 µg/mL) as the internal standard; to this, 100 µL of sodium bicarbonate pH 9.5 was added, after which methanol (LC-MS grade) was incorporated to bring the remaining volume up to 1 mL. Samples were place on vortex for 30 s, then placed on a mechanical shaker for 10 min, and centrifuged for 15 min at 130 × 100 RPM. The supernatant layer was collected and filtered with 0.22 µ PTFE filter and transferred to HPLC vial and then injected using the previous chromatographic conditions. Benzodiazepines were identified, and their concentrations were calculated using the corresponding regression equations.

## 4. Conclusions

The analytical method was initially developed using HPLC-UV instrumentation. To the best of our knowledge, it represents the shortest validated method for the simultaneous determination of benzodiazepine mixtures while maintaining acceptable levels of accuracy, precision, sensitivity, and selectivity. This study presents a rapid and accurate method for the simultaneous determination of Alprazolam, Bromazepam, Clonazepam, Diazepam, and Flunitrazepam in human urine. The analysis was performed on a C18 column using reverse-phase chromatography coupled with LC-MS/MS, and its applicability was demonstrated on samples obtained from those suspected of drug abuse.

The developed method features a shorter run time and produces sharp, distinct peaks, contributing to its rapidity and accuracy. The sample preparation involves a straightforward liquid–liquid extraction procedure. All validation parameters met the acceptance criteria, confirming the method’s sensitivity, precision, accuracy, and linearity over the tested concentration range. Application of this method to 48 urine samples from those suspected of drug abuse in the Kuwaiti region resulted in a drug-positive identification rate of 93.75%, with Bromazepam and Alprazolam being the most commonly detected substances, followed by Clonazepam and Diazepam. This study demonstrates that the developed and validated method is suitable for routine laboratory analysis of Alprazolam, Bromazepam, Clonazepam, Diazepam, and Flunitrazepam in human urine samples, including quality control samples.

Future work could extend this approach to samples from other geographic locations, such as Canada, where benzodiazepines were detected in 11.3% of cases in the Nova Scotia region [45], and involve larger sample sizes to broaden epidemiological insights. Additionally, cross-validation using different biological forensic matrices and comparison between LC-MS and GC-MS data could enhance the method’s robustness and applicability for wider forensic investigations.

Although matrix effects were not directly quantified through post-extraction spiked samples, the method was developed with careful attention to minimizing ion suppression and enhancement via optimized sample preparation and chromatographic separation. Consistent analyte recovery and precision across multiple synthetic urine batches suggest minimal matrix interference under the tested conditions. We acknowledge that direct assessment of matrix effects in authentic urine samples would further strengthen the method, and such evaluations are planned for future studies.

In the future, if this method is subjected to the analysis of human urine samples in forensic cases for various benzodiazepines and its analogues and antipsychotic or different chemical classes such as opiates, cocaine, and amphetamines, then high-throughput dispersive liquid/liquid microextraction (DLLME) could be adopted using LC-MS/MS with triple quadrupole or ion trap technology for targeted compounds, while for non-targeted compounds, high-resolution mass spectrometry using orbitrap or time of flight mass spectrometer could be adopted.

## Figures and Tables

**Table 1:** Results of within-run accuracy and precision for alprazolam, bromazepam, clonazepam, diazepam andflunitrazepam.

Concentration in ng/mL.	Bromazepam			Clonazepam		
	% Mean Recovery	Standard Deviation	% RSD	% Mean Recovery	Standard Deviation	% RSD
	of the Calculated Result (*n* = 3)			of the Calculated Result (*n* = 3)		
2.0	118.188	3.968	3.372	111.044	7.413	6.676
6.0	100.870	3.367	3.338	104.750	5.501	5.252
100.0	112.253	1.484	1.322	94.961	1.351	1.422
200.0	107.795	3.692	3.425	93.734	1.277	1.363
	**Alprazolam**			**Flunitrazepam**		
2.0	98.156	4.116	4.193	100.249	9.482	9.459
6.0	96.520	3.422	3.545	107.440	7.015	6.529
100.0	105.240	1.459	1.387	82.356	5.621	6.825
200.0	103.905	2.494	2.400	86.120	0.707	0.821
		**Diazepam**				
2.0	104.655	7.914	7.562			
6.0	98.591	6.189	6.277			
100.0	105.282	2.427	2.305			
200.0	105.407	2.266	2.149			

**Table 2:** Results of precision for %RSD of peak area ratio.

Name of Analyte.	Concentration in ng/mL	Repeatability	Intermediate Precision
		% RSD of Peak Area Ratio	
Bromazepam	100.0	0.82	1.85
Clonazepam		1.34	3.05
Alprazolam		1.00	2.38
Flunitrazepam		0.95	2.43
Diazepam		0.82	1.21

The results obtained for this entire study were calculated using Excel, Microsoft 365.

**Table 3:** Analysis of benzodiazepines in street samples in Kuwait using the proposed LC-MS/MS method.

Serial No.	Sample No.	Bromazepam	Clonazepam	Alprazolam	Flunitrazepam	Diazepam
		(Average Concentration in Triplicate (ng/mL))				
1	sam46	5.4748	1.2251	6.0697	-	2.1709
2	sam47	5.4886	2.2427	3.9326	-	3.9460
3	sam48	4.9436	0.4650	4.5253	-	2.2870
4	sam49	7.2516	-	5.7366	-	2.5102
5	sam50	5.0215	0.6228	5.3686	-	2.0624
6	sam 51	5.2696	0.6858	5.2520	-	3.3663
7	sam52	5.0583	0.8532	4.5659	-	2.8131
8	sam53	5.3205	1.0782	12.5619	2.2799	2.7165
9	sam54	5.0251	1.1383	4.7316	-	2.4005
10	sam55	-	-	-	-	-
11	sam56	5.0142	1.2450	4.4569	3.6955	6.3266
12	sam57	5.0805	0.5725	5.1489	-	4.9194
13	sam58	5.3646	0.9026	5.5856	-	1.9269
14	sam59	9.7299	2.6409	4.5594	-	1.4427
15	Sam60	5.0183	1.0396	5.3341	-	1.9927
16	sam61	5.1545	0.9852	5.5671	-	1.9981
17	sam62	6.2667	0.9860	4.9319	-	3.4057
18	sam63	6.1029	1.2010	6.2537	-	2.4384
19	sam64	4.9545	9.1650	4.6334	5.4524	13.6228
20	sam65	6.0903	10.0788	5.9130	6.0009	12.7135
21	sam66	5.2878	0.5787	4.9598	-	9.8523
22	sam67	6.7173	0.9566	4.5280	-	2.2346
23	sam68	-	-	-	-	-
24	sam 69	5.7848	1.2066	6.0432	-	12.3481
25	Sam 70	5.1985	1.9877	6.4398	3.0140	-
26	Sam71	4.9441	--	5.2073	-	14.0141
27	sam 72	6.9293	0.7251	5.9935	2.9429	3.2799
28	sam 73	6.0371	1.9585	2.2617	-	5.8814
29	sam74	4.9269	0.5910	4.4989	-	5.2207
30	sam75	5.1447	1.2810	2.5473	-	7.7373
31	sam 76	5.1885	0.6395	4.3771	-	8.4144
32	sam77	5.2606	1.2079	233.7426	-	3.1451
34	sam 78	5.0248	0.5873	4.7209	2.5989	2.3015
35	sam79	5.1484	0.5479	5.2195	-	1.9525
36	sam 80	5.2637	0.8489	5.0545	-	2.0228
37	sam 81	5.1437	0.7519	6.1830	-	2.1318
38	sam82	4.9497	0.7275	99.7967	-	2.4242
39	sam 83	4.9711	0.6097	5.9817	-	2.2114
40	sam 84	5.1864	0.5213	6.2792	-	2.1758
41	sam85	4.9337	1.1259	2.2515	-	2.7399
42	sam86	5.2481	0.8119	1.8735	-	2.7906
43	sam 87	-	-	-	-	-
44	Sam88	5.0336	8.3261	4.6716	-	4.0663
45	sam 89	8.2721	2.2338	4.7494	-	11.3568
46	sam 90	5.0125	-	5.0546	-	10.4125
47	sam 91	5.0383	0.7838	4.5123	-	5.6242
48	sam 92	4.9101	0.7944	178.9956	-	2.4911

‘-’ indicates no traces observed.

**Table 4:** Gradient for chromatographic separation.

Time in Minutes	% Composition of B
0.01	30
1.00	30
10.00	100
12.00	100
12.10	30
15.00	30

**Table 5:** Multiple reaction monitoring (MRM) transitions, retention time (minutes) and collision energy (CE).

Compound	Retention Time (minutes)	Precursor Ion (*m/z*)	Product Ion (*m/z*)	Collision Energy (CE)
Bromazepam	6.172	317.95	209.05	−28.0
			182.05	−33.0
Clonazepam-D4	7.520	320.25	274.20	−26.0
			218.10	−39.0
Clonazepam	7.558	316.05	270.10	−25.0
			214.00	−37.0
Alprazolam	7.818	309.10	281.15	−26.0
			205.15	−45.0
Flunitrazepam	8.120	314.10	268.10	−25.0
			239.15	−36.0
Diazepam	9.247	285.10	193.15	−30.0
			154.10	−27.0

## Data Availability

Data set available on request from the authors.
